# Enumeration optimization of open pit production scheduling based on mobile capacity search domain

**DOI:** 10.1038/s41598-022-27336-y

**Published:** 2023-01-03

**Authors:** Xiaochuan Xu, Xiaowei Gu, Qing Wang, Yunqi Zhao, Wenyuan Kong, Zhenguo Zhu, Fengdan Wang

**Affiliations:** 1 College of Resources and Civil Engineering, Northeastern University, Shenyang, China; 2 Science and Technology Innovation Center of Smart Water and Resource Environment, Northeastern University, Shenyang, 110819 China

**Keywords:** Environmental sciences, Mathematics and computing

## Abstract

The optimization of open pit mine production scheduling is not only a multistage decision-making problem but also involves space–time dynamic action among multiple factors, which makes it difficult to optimize production capacity, mining sequence, mining life, and other factors simultaneously in optimizing design. In addition, the production capacity is disorderly expanded, the calculation scale is large, and the optimization time is long. Therefore, this article designs a mobile capacity search domain method to improve computing efficiency without omitting the optimal production capacity. At the same time, taking the maximum net present value as the objective function, an enumeration method is used to optimize the possible paths in different capacity domains and calculate the infrastructure investment and facility idle cost required to meet the maximum production capacity on each possible path to control the disorderly expansion and violent fluctuation of production capacity. The research shows that the open pit mine production scheduling optimization algorithm proposed in this article can not only realize the simultaneous optimization of the three elements of production capacity, mining sequence, and mining life but also improve the computing efficiency by 200 times. Furthermore, the production capacity fluctuation is less than 1.4%. The mining life of the mine is extended by 13 years, and the overall economic benefit is increased by 18%.

## Introduction

The production capacity, mining sequence and mining lifetime are the three significant factors in the optimization of open pit mine production scheduling. While production capacity is related to technical conditions of the mine, mining sequence is related to mine organization management, and mining lifetime is restricted by mine scheduling and industry norms; furthermore, these three factors are affected by geological conditions of the ore body, reserves scale, marketing environment and other elements^1–4^. In addition, compared with other external conditions, there is also interaction and influence among the three elements of production scheduling. For example, the more significant the production capacity is, the shorter the mining productive life. Different mining sequences (pit advancement position at the end of the year) directly affect the year's production capacity and then affect the overall mining life. In practice, therefore, optimized mining scheduling determines the annual estimated amount of mined ore, the annual estimated amount of stripped waste rock (i.e., production capacity), which areas are mined and stripped each year or how each step is advanced (i.e., the mining sequence), and the mining period (i.e., the mining lifetime).

Production scheduling provides a future production strategy for a mine (new mine or mine in producing). Moreover, for a given deposit, the quality of the production schedule has a significant impact on infrastructure investment and cash flow, which is distributed on the timeline after production. This influence becomes an important factor in the investment return rate of the whole mine project. That is why international mining companies have a strong interest in optimizing production scheduling. Production scheduling optimization is always a popular research topic in mine system engineering. From the perspective of optimization, the open pit mine production schedule determines the mining time of each module in the massive deposit model to determine which module should be mined each year to maximize the total NET present value and meet the space–time relationship and technical and economic constraints of open pit mining.

The "mining increment sorting method" is the earliest computer optimization method applied to production scheduling. It was first proposed by engineers of the Kennecott Company and applied in the company. The mining increment is generated by constructing cones, and cone structure, evaluation and sequencing are carried out via human–computer interaction trial and error^5^. Production increments for production scheduling can be obtained by the "parametric analysis" proposed by Lerchs and Grossmann^6^. This method is further developed into the "reserve parameterization" method, and many scholars have carried out further research on the solution of reserve parameterization and its application in production scheduling^7–12^.

One of the inherent defects of parameterization is the "notch" problem; in the generated limit sequence, the increments between some of the adjacent limits are so large that the limit sequence cannot be used for production scheduling optimization. Therefore, some researchers use heuristic algorithms to generate nested limit sequences to overcome the gap problem^13–20^ and dynamically order the resulting limit sequence^18,21–25^.

In summary, the essence of the production scheduling optimization problem is to determine the optimal production time of each module on the premise of meeting the necessary constraint conditions to obtain the maximum total NET present value. It is a typical linear programming problem. Therefore, linear programming (its specific form includes mixed programming, pure integer programming and 0–1 programming) is one of the most commonly used mathematical optimization methods to solve the optimization problem of production scheduling; related studies were carried out as early as the late 1960s ^26,27^. Many researchers have established linear programming models with different concrete forms for different aspects of production scheduling problems^27–35^.

However, the quantity of variables and constraint equations in the linear programming model for optimizing production scheduling is too enormous when a single module in the massive deposit model is used as the decision unit. That is a situation that even today's computers cannot solve directly; if it is integer programming, it can hardly be solved. Therefore, some researchers try to find solutions in the construction of mathematical model forms (mainly constraints) or solving algorithms (usually by feat of approximate algorithms) to boost the solving speed^36–42^. Increasing the decision-making unit to reduce the quantity of variables and constraints is a more common way, such as combining the modules in the deposit model into a "unit tree" as the decision unit in optimization or taking steps or panels as the decision unit^37,43–51^. However, due to the low scheduling accuracy (or resolution) in enormous decision units, the results are significantly different from the optimal plans, which also reduces the practicality of the results^47,49,52,53^. Therefore, many researchers make use of the unique structure of the mathematical model to reduce the model size with the Lagrange relaxation method and solve the model with other measures and algorithms, such as iteration, decomposition, gradient method, and the Dantzig network flow method^54–68^. The biggest obstacle to this approach is the "gap" problem. Researchers have tried various methods to solve this problem, but they have not sought out suitable means.

Open pit mine production scheduling is a typical multi-period decision-making problem; moreover, the time is interrelated so that dynamic programming can be used to solve it. Therefore, dynamic programming is also one of the most used mathematical optimization methods to solve the problem. Researchers have used dynamic programming to study the optimization of different aspects of production scheduling^1,69–84^.

Because of the insurmountable difficulties in applying mathematical optimization models to obtain the exact solution of production scheduling, some researchers turn to approximate algorithms, such as genetic algorithms, random, local search, particle swarm algorithms, and simulations, to obtain one or more "perfect" plans^85–97^.

In the process of production schedule optimization, in addition to exploring mathematical methods to solve the problem, another factor is to consider the specific influencing factors of the optimization process. In the study of the traditional optimization problem, first, it is assumed that a known factor could affect the optimization of the production schedule. For example, most studies determine the mine production capacity according to the Taloy formula, similar mine factors, or relevant experience and then optimize the mining sequence based on the determined production capacity. Finally, the relevant infrastructure is allocated according to the production scheduling^98–101^ set boundary grades in different periods with the maximum net present value as the objective function to ensure that the infrastructure meets the maximum comprehensive processing capacity of mining, beatification and smelting. This method can obtain higher economic benefits than the Lane method; nonetheless, the characteristic of uniform grade distribution is not applicable to most mines. On the side, the method described by Khan and Asad et al. focuses on the impact of geological uncertainty, marketing environment change and risk management on production scheduling^83,101–108^.

Consequently, due to the high complexity of production scheduling optimization and the difficulty of finding an exact solution, it is still a popular research topic. Many problems in the process of production scheduling optimization of open pit mines exist, such as low operation efficiency caused by large amount of calculation, disorderly expansion of production capacity caused by the scale effect, and difficulties in simultaneously optimizing the three elements due to the interaction relationship. In view of these problems, taking the maximum comprehensive net present value (NPV) as the objective function, the method of mobile capacity search domain is proposed to improve the operation efficiency, the infrastructure investment function based on the maximum production capacity is constructed to restrict the production capacity, the facility idle threshold is designed to reduce the fluctuation range of production capacity, and the enumeration method is used to evaluate all workable paths to realize the simultaneous optimization of the production capacity, mining sequence and mining life of open pit mines.

### Mathematical model of discrete body dynamic programming

The purpose of production scheduling optimization of open pit mines is to determine how much ore and rock are extracted and stripped every year, where they are advanced and how long the mining life is. Therefore, the deposit should be divided into a finite number of discrete bodies, which should be taken as the alternative objects of open pit mining at the end of the year. Due to the characteristics of open pit mining technology and safety requirements, mining engineering needs to be carried out gradually from top to bottom in accordance with the specified slope angle, and the division of discrete bodies needs to meet the geometric space relationship. According to the definition and generation principle of the "geological optimal pit" proposed by^1^, a series of fully nested discrete bodies, namely, the "geological optimal pit sequence", can be obtained. The specific process for generating the "geological optimal pit sequence" is as follows:

Started from the bottom of the ultimate pit (also the first geological optimal pit), floating cone exclusion method is used to find out all the parts whose ore volume is equal to the given increment (such as 2 million tons), and among these parts, the one that holding the lowest average grade removed from the ultimate pit, leaving a pit that is 2 million tons less than the ultimate pit. The pit has the highest metal content of all the pits, with 2 million tons less ores than the ultimate pit. So the second geological optimal pit (the first is the ultimate pit) is obtained. Then, in the remaining pit (the second geological optimal pit), remove the part with the ore volume equal to the given increment (2 million tons) and the minimum average grade by the same method, and a smaller pit (the third geological optimal pit) is obtained. According to the method analogy, the ultimate pit is discretized into a series of completely nested discrete body sequences, that is to say, the geological optimal pit sequence.

**Figure 1:**
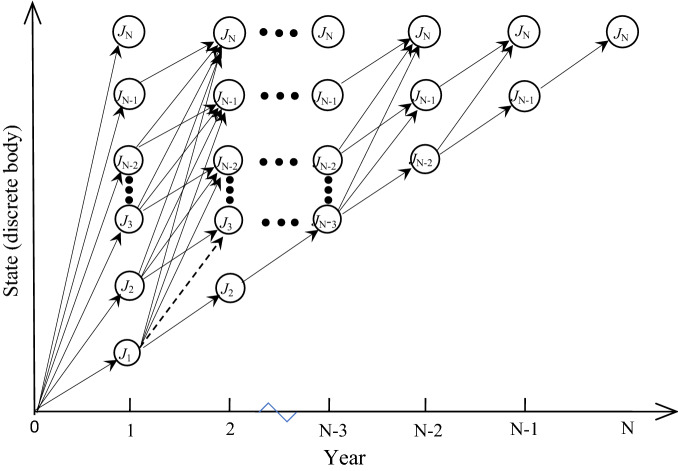
Discrete body dynamic sorting.

The discrete bodies are put into the dynamic sorting model as state variables, as shown in Fig. 1.

The circles in Fig. 1 represent a discrete body (geological optimal pit), which is also where the pit is advancing at the end of a year. The two circles linked by arrows, such as J_1_ in the first year and J_3_ in the second year, linked by dotted arrows, indicate that the open pit advances to J_1_ in the first year and J_3_ in the second year. In geometric space, J_3_ completely contains J_1_, therefore the quantity of ore corresponding to J_1_ and J_3_ can be expressed as O_1_,_1_ and O_2_,_3_, and the quantity of rock can be expressed as R_1,1_ and R_2,3_. In fact, since the quantities of ore and rock of each discrete body have been determined, O_1,1_ and O_2,3_ are equal to O_1_ and O_3_; R_1,1_ and R_2,3_ are the same as R_1_ and R_3_, mainly for the purpose of relating to time. Then, the quantity of ore and rock of the *j*th discrete body corresponding to the *i*th year can be expressed as O_*i,j*_ and R_*i,j,*_ respectively. Therefore, the quantity of ore *o*_*i*−1, *k*_ (*i, m*) and rock produced from discrete body J_*k*_ in year *i − *1 to discrete body J_*m*_ in year *i* where *m* > *k*, because the discrete body must be advanced from small to large, are *r*_*i−*1*,k*_ (*i, m*), respectively:

1 $$o_{i - 1,k} \left( {i,m} \right) = \, ({\text{O}}_{{i - {1},k}} - {\text{O}}_{i,m} ) \times \gamma /\left( {{1} - \delta } \right)$$

2 $$r_{{i - {1},k}} \left( {i,m} \right) = \, \left( {{\text{R}}_{{i - {1},k}} - {\text{ R}}_{i,m} } \right) - o_{{i - {\text{1,k}}}} \left( {i,m} \right) \, \times \delta + \, \left( {{\text{O}}_{{i - {1},k}} - {\text{ O}}_{i,m} } \right) \times \left( {{1} - \gamma } \right)$$

where γ is the ore recovery rate and δ is the mixing rate of waste rock.

The concentrate amount *v*_*i*-1_,_*k*_(*i*, *m*), which transitions from the discrete body J_*k*_ in year *i − *1 to the discrete body J_*m*_ in year *i,* is:

3 $$v_{i - 1,k} \left( {i,m} \right) = \left[ {\left( {O_{i - 1,k} \times g_{k} - \, O_{i,m} \times g_{m} } \right) \times \gamma + o_{i - 1,k} \left( {i,m} \right) \, \times \delta \times g_{r} } \right]/g_{v}$$

where *g*_*k*_ and *g*_*m*_ represent the average grade of ore in the *k*th and *m*th discrete bodies; *g*_*r*_ represents the average grade of rock (R_*i-*1*,k*_ − R_*i,m*_) transiting from discrete J_*k*_ to discrete J_*m*_; *g*_*v*_ refers to concentrate grade.

The profit *u*_*i* − 1_,_*k*_(*i*, *m*), which transitions from the discrete body J_*k*_ in year *i* − 1 to the discrete body J_*m*_ in year *i,* is:

4 $$u_{i - 1,k} \left( {i,m} \right) = v_{i - 1,k} \left( {i,m} \right) \, \times p_{v} \times \left( {1 + \eta } \right)^{i - 1} - \left[ {o_{i - 1,k} \left( {i,m} \right) \, \times \left( {c_{o} + c_{p} } \right) \, \times \left( {1 + \varepsilon } \right)^{i - 1} + r_{i - 1,k} \left( {i,m} \right) \, \times c_{r} \times \left( {1 + \varepsilon } \right)^{i - 1} } \right]$$

where *p*_*v*_ refers to the price of concentrate, ¥/t; *c*_*o*_, *c*_*p*_ and *c*_*r*_ represent mining cost, processing cost and stripping cost, respectively, ¥/t; and *η* and *ε* represent the price rise rate and cost rise rate, respectively.

The net present value NPV_*i*-1_,_*k*_ (*i*, *m*), which transitions from the discrete body J_*k*_ in year *i* − 1 to the discrete body J_*m*_ in year *i,* is:

5 $${\text{NPV}}_{{i - {1},k}} \left( {i,m} \right) \, = u_{{i - {1},k}} \left( {i,m} \right)/\left( {{1} + \lambda } \right)^{i}$$

where *λ* represents the discount rate.

The transition process mentioned above is the transition process between different states. Without considering the constraints of production capacity, any year in Fig. 1 may be the final life of the mine. It is also the year in which the mine is mined to the final state J_N_ (discrete body, which is also the ultimate pit of the open pit mine). In addition to the three production costs of mining, stripping and processing given in Eq. (4), another significant cost is infrastructure investment. It generally occurs at the initial stage of mine construction, i.e., the infrastructure investment does not affect the state transition shown in Fig. 1. However, it will affect the final economic benefit of the mine. Therefore, it is necessary to consider the infrastructure investment in each NPV that reaches the final state J_N_ through different paths. Although infrastructure investment has no influence on a state transition, infrastructure investment is affected by mine production capacity, and mine infrastructure investment usually needs to meet the maximum production capacity in the whole life cycle of the mine. In other words, the infrastructure investment needs to meet the maximum ore quantity difference between any two adjacent time points in the state transition process on a path to the final state J_N_, as shown in Fig. 1; that is, the maximum production capacity on this path is max{*o*_*i-1,k*_(*i, m*)}. According to Eq. (1), the quantity of ore produced by state transition at two adjacent time points is *o*_*i*-1_,_*k*_(*i*, *m*). Therefore for a particular path L(D) with a mining life of D years, its maximum production capacity q_L(D)_ is:

6 $$q_{L\left( D \right)} = \mathop {max}\limits_{{k \in \left[ {i - 1,m - 1} \right]}} \left\{ {{ }\left( {{\text{O}}_{i - 1,k} { } - {\text{ O}}_{i,m} { }} \right) \times {\upgamma }/\left( {1 - {\updelta }} \right){ }} \right\}$$

The infrastructure investment of the mine can be approximated as a linear function of the maximum production capacity of the ore. For the path L(D) with a mining life of D years, the infrastructure investment *c*_L(D)_ can be approximated as:

7 $$c_{{{\text{L}}({\text{D}})}} = {\text{a}} + {\text{b}} \times q_{{{\text{L}}({\text{D}})}}$$

where *a* refers to the infrastructure investment base unrelated to the production scale, 10^4^ ¥, and b refers to the infrastructure investment per unit of mining amount, ¥/t.

The comprehensive economic benefit NPV_L(D)_ for path L(D) can be expressed by the following formula:

8 $${\text{ NPV}}_{{{\text{L}}\left( {\text{D}} \right)}} = c_{{{\text{L}}\left( {\text{D}} \right)}} + \mathop \sum \limits_{i = 1}^{D} {\text{NPV}}_{i - 1,k} \left( {i,{ }m} \right).$$

### Enumeration optimization algorithm of production scheduling

Set a wide enough range of ore production capacity [*q*_*low*_, *q*_*up*_], and the optimal production capacity must be within this range. This range is set by the user and is the input data. Let *n* represent the ordinal number of the constraint domain of ore production capacity, and [*q*_L_^*n*^, *q*_U_^*n*^] define the *nth* constraint domain (capacity search domain).

- **Step one:** Set n = 1 and juxtapose the Bohr variable LastPlan = false. In the discrete body sequence {J}_N_, the discrete body whose ore quantity is greater than or equal to and closest to *q*_*low*_ is found, and its number is denoted as H.
- **Step two:** Set the lower bounds *q*_L_^*n*^ and upper bounds *q*_U_^*n*^ of the constraint domain [*q*_L_^*n*^, *q*_U_^*n*^]:
  9 $$q_{{\text{L}}}^{n} = {\text{O}}_{{\text{H}}} {-}\Delta {\text{P}}/{2}$$
  where ΔP is the ore increment of the pit set when the discrete body sequence {J}_N_ is generated^1^, and O_H_ is the ore quantity of the Hth discrete body in the sequence {J}_N_.10where O_N_ is the total ore quantity in the ultimate pit and *x*_1_, *x*_2_, *x*_3_ are the search domain constraints, which are determined according to the size of the mine. If G > N obtained by Eq. (10), set G = N. The upper bound of the domain is
  11 $$q_{{\text{U}}}^{n} = {\text{O}}_{{\text{G}}} + \Delta {\text{P}}/{2}$$
  where O_G_ is the ore quantity of the Gth discrete body in sequence {J}_N_.
  If *q*_U_^*n*^ > *q*_*up*_, LastPlan = true.
- **Step three:** Find all production scheduling whose annual ore output meets the constraints of the domain [*q*_L_^*n*^, *q*_U_^*n*^]. That is, find all subsequences in which the annual ore output falls into the domain [*q*_L_^*n*^, *q*_U_^*n*^]. Calculate the NPV of each schedule, record the schedule with the highest NPV, and call the schedule the "local best schedule" in the domain [*q*_L_^*n*^, *q*_U_^*n*^]. The specific method is as follows:
  - **Step 1:** Set *i* = 1 (first year). A discrete body is found in the discrete body sequence {J}_N_. The ore quantity is no less than and closest to *q*_L_^*n*^, the lower limit of the constraint domain [*q*_L_^*n*^, *q*_U_^*n*^] of the set ore production capacity, and the total quantity of ore and rock is no more than *q*_*up*_ of the set annual mining and stripping capacity. If such a discrete body is found, its number in {J}_N_ is H(1); that is, the pit at the end of the first year on the planned path L under construction is advanced to pit J_H(1)_*. According to Eqs. (1), (3), calculate the quantities of produced ore *o*_1_, concentrate *v*_1_ and stripped waste rock *r*_1_ in this year. Then, calculate the annual profit *u*_1_ and its net present value NPV_1_ discounted to time 0 according to Eqs. (4), (5). Move on to the next step. If such a discrete body is not found, there is no workable schedule, and the algorithm terminates.
  - **Step 2:** set *i* = *i* + 1 (next year).
  - **Step 3:** The discrete body number H(*i*) is equal to H(*i* − 1) + 1 in year *i*, and H(*i*-1) is the discrete body number of the previous year of the planned path L under construction.
  - **Step 4:** According to Eqs. (1), (2), calculate the ore mining quantity *o*_*i*_ and waste rock stripping quantity *r*_*i*_ from *i* − 1 to *i*.
  - **Step 5:**
    (A) If *o*_*i*_ < *q*_L_^*n*^ and *o*_*i*_ + *r*_*i*_ ≤ *q*_*up*_.
    (B) H(*i*) = N, that is, the discrete body J_H(*i*)_ is the last (ultimate pit) in the sequence {J}_N_, then the planned path L under construction has reached the end point, and the final state J_N_ is the discrete body of the end point on the path. A complete workable planned path is obtained, and its mining life D = *i*. According to Eq. (3), the concentrate quantity *v*_*i*_ in the first year is calculated, and then according to Eq. (4–5), the annual profit *u*_*i*_ and its net present value NPV_*i*_ discounted to time 0 are calculated. Go to step 6.
    (C) H (*i*) < N, the ore quantity of year *i* is lower than the lower limit of the set annual ore production capacity, which is not workable. Set the discrete body number H(*i*) to H(*i*) + 1, that is, consider a larger discrete body, and return to step 4.
    (D) *q*_L_^*n*^ ≤ *o*_*i*_≤ *q*_U_^*n*^and *o*_*i*_ + *r*_*i*_ ≤ *q*_*up*_.
      Discrete body J_H(*i*)_ is the workable pit state at the end of *i* on the planned path L under construction. According to Eq. (3), the concentrated quantity *v*_*i*_ of year *i* was calculated. Then, according to Eqs. (4), (5), the annual profit *u*_*i*_ and its net present value NPV_*i*_discounted to time 0 were calculated. If H(*i*) = N, then the planned path under construction has reached the final state, and a complete workable planned path has been obtained. Set the mining life of the intended path to D = *i* and carry out step 6. Otherwise, return to step 2.
    (E) If *o*_*i*_ > *q*_U_^*n*^ or *o*_*i*_ + *r*_*i*_ > *q*_*up*_.
      The quantity of ore mining or the total quantity of mining and stripping in year *i* exceeds the set upper limit, there is no workable plan, and the algorithm terminates.
  - **Step 6:** Thus far, a workable planned path with "minimum ore output" is obtained, that is, the quantity of ore extracted in each year of the path except the last year is just enough to meet the set minimum annual ore production capacity *q*_L_^*n*^. Calculate the infrastructure investment *c*_L(D)_ of the shcedule according to Eqs. (6), (7). Calculate the total NPV _L(D)_ of this path according to Eq. (8). Take the path as the current path and save it as the best path.
  - **Step 7:** Set time *i* = D − 1, where D is the mining life of the current path L.
  - **Step 8:** Build a new workable plan path starting from year *i*. The new path will be the same as the current path in year 1 to (*i* − 1). Add 1 to the current path's discrete body number of year *i*, that is, set H(*i*) = H(*i*) + 1.
  - **Step 9:** According to Eqs. (1), (2), calculate the ore quantity *o*_*i*_ and waste rock quantity *r*_*i*_ from *i* − 1 to *i*.
  - **Step 10:** Distinguish feasibility and economic evaluation, divided into two cases:
    (A) If *o*_*i*_< *q*_U_^*n*^ and *o*_*i*_ + *r*_*i*_ ≤ *q*_*up*_*.*
      Discrete body J_H(*i*)_ is the workable pit state at the end of year *i* of the new planned path being constructed, and it becomes the discrete body of last year *i* of the current path (that is, the original discrete body is replaced). According to Eq. (3), the concentrated quantity *v*_*i*_ of year *i* is calculated. Then, according to Eqs. (4), (5), the annual profit *u*_*i*_ and its net present value NPV_*i*_discounted to time 0 are calculated. If H(*i*) = N, the new planned path under construction has reached the final state and a complete workable planned path has been obtained, and its mining life is D = *I*; go to step 15. Otherwise, go to step 11.
    (B) If *o*_*i*_ > *q*_U_^*n*^ or *o*_*i*_ + *r*_*i*_ > *q*_*up*_.
      If the quantity of ore mining or the total quantity of mining and stripping in year *i* exceeds the upper limit set, it is not workable. Set *i* = *i* − 1, that is, go back one year along the current path. If *i* > 0, go back to step 8. Otherwise, all workable plan paths are constructed and evaluated; proceed to step 16.
  - **Step 11:** Set *i* = *i* = *i* + 1.
  - **Step 12:** The discrete body number H(*i*) is equal to H(*i* − 1) + 1 in year *i*, where H(*i*-1) is the discrete body number of the previous year of the new planned path being constructed.
  - **Step 13:** According to Eqs. (1), (2), calculate the ore quantity *o*_*i*_ and waste rock stripping quantity *r*_*i*_ from *i* − 1 to *i*.
  - **Step 14:**
    (A) if *o*_*i*_< *q*_L_^*n*^ and *o*_*i*_ + *r*_*i*_ ≤ *q*_*up*_, in two cases:
    (B) H(*i*) = N, that is, the discrete body J_H(*i*)_ is the last (ultimate pit) in the sequence {J}_N_, the new planned path under construction has reached its endpoint, and the ultimate pit J_N_ is the end discrete body on this path. Finally, a complete workable planned path is obtained, and its mining life D = *i*. According to Eq. (3), the concentrated quantity *v*_*i*_ of year *i* was calculated, and then according to Eqs. (4), (5), the annual profit *u*_*i*_ and its net present value NPV_*i*_ discounted to time 0 were calculated. Perform step 15.
    (C) H(*i*) < N, the ore quantity of year *i* is below the lower limit of the set ore production capacity, which is not workable. Set discrete body number H(*i*) = H(*i*) + 1, that is, consider a larger mining discrete body, and return to step 13.
      If *q*_L_^*n*^ ≤ *o*_*i*_≤ *q*_U_^*n*^ and *o*_*i*_ + *r*_*i*_ ≤ *q*_*up x*_.
      Discrete body J_H(*i*)_ is the workable state at the end of year *i* on the new plan path under construction. According to Eq. (3), the concentrated quantity *vi* of year *i* was calculated, and then according to Eqs. (4), (5), the annual profit *u*_*i*_ and its net present value NPV_i_ discounted to time 0 were calculated. If H(*i*) = N, then the new planned path under construction has reached the final state, and a complete workable planned path has been obtained. Its mining life is D = *i*, and step 15 is carried out. Otherwise, return to step 11.
    (D) If *o*_*i*_ > *q*_U_^*n*^ or *o*_*i*_ + *r*_*i*_ > *q*_*up*_.
      The quantity of ore mining or the total quantity of mining and stripping in year *i* exceed the set upper limit, which is not workable. The algorithm gives up halfway and displays error information. Go to step 16 and output the best path thus far.
  - **Step 15:** After a new workable scheduling path is established, calculate the infrastructure investment *c*_L(D)_ of the schedule according to Eqs. (6), (7). Calculate the total NPV _L(D)_ of this path according to Eq. (8). If the total NPV _L(D)_ of this path is greater than the total NPV _L(D)_ of the saved best path, the path is reserved as the best path (that is, the original best path is replaced); otherwise, the original optimal path remains unchanged. Take this path as the current path and return to step 7.
  - **Step 16:** Output the best-planned path on the interval [*q*_L_^*n*^, *q*_U_^*n*^].
- **Step four:** If LastPlan = false, go to the next step. Otherwise, if LastPlan = true, go to step six.
- **Step five:** Set *n* = *n* + 1 and H = H + 1. Return to step two and set the next constraint domain, and continue the iteration. In this iteration process, every time H increases by 1, the lower bound *q*_*L*_^*n*^ of the domain calculated according to Eq. (9) increases once. The whole domain also moves to a higher production capacity.
- **Step six:** The constraint domain covers the entire range of ore production capacity [*q*_*low*_, *q*_*up*_]. The best schedule is obtained by finding the one with the highest NPV among all the local best schedules recorded; Output the best schedule. The algorithm can also output all local best schedules to see how NPV varies with ore production capacity. The algorithm ends.

**Figure 2:**
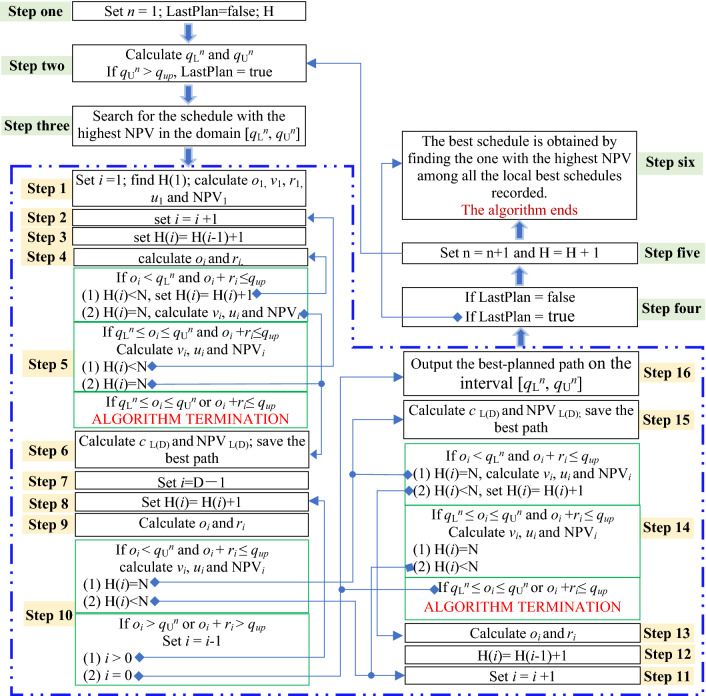
Flow chart of enumeration optimization algorithm for production scheduling.

In the above algorithm, the larger the width of the constraint domain (*q*_L_^*n*^, *q*_U_^*n*^) is, the more schedules satisfy the constraint of (*q*_L_^*n*^, *q*_U_^*n*^). If the domain is set wide, the number of schedules to be evaluated in step three is too large and time-consuming. If the range is too narrow, the probability of missing the optimal schedule is high. The conditions listed in Eq. (10) in step two are to control the width of the constraint domain within such a range. In addition, for a given domain width, the number of schedules s satisfying the domain constraint increases rapidly with increasing mining life. The detailed algorithm flow is shown in Fig. 2.

Among the workable scheduled paths, the difference between some paths' total NPV and maximum total NPV is very small, which can be ignored. However, some paths may be more reasonable than those with the maximum total NPV, such as more stable ore yield and lower peel peak. Therefore, in the above algorithm, multiple optimal paths can be reserved and output for users to choose. When designing optimization software, the number of optimal paths to be reserved should be set by users as input data on the interface.

## Case application

### Basic introduction of mining area

**Figure 3:**
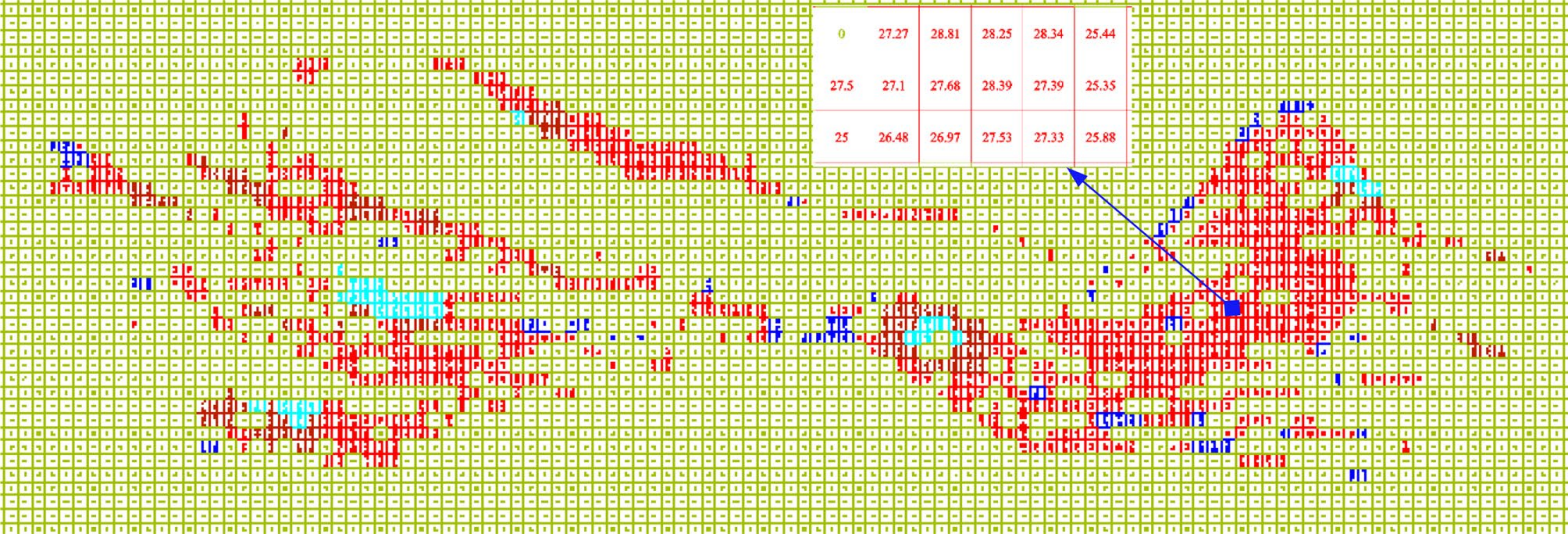
Grade distribution at − 172 m.

The research takes the example of a large open pit iron mine in Luanzhou City, Hebei Province. The mine started construction in 2004 and reached production in 2009. The mining area has reserves of 2.3 billion tons with an average grade of 28.13% (Fig. 3). The details of this mine and the raw date for algorithm can be get in Supplementary Information.

**Figure 4:**
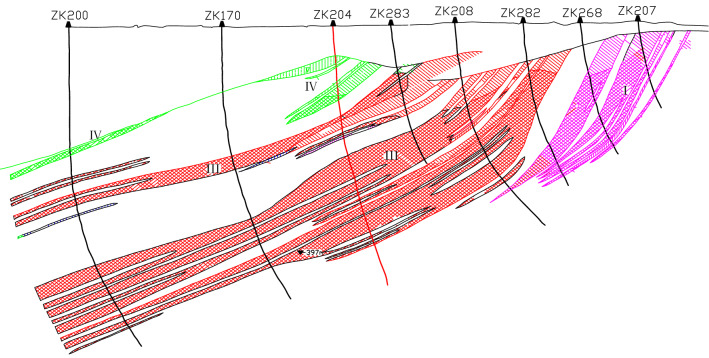
Section of exploration Line 12.

There are four orebodies distributed from east to the west in a parallel zonal arrangement. Figure 4 shows the section corresponding to exploration Line 12. The No. 1 ore-body is located in the east of the mining area, running through the three areas of north, central and south, with a total length of 8350 m and an average thickness of 60 m. The No. 2 ore body is located in the middle of the mining area, with a total length of approximately 900 m and an average thickness of 45 m. The ore body has a large extension depth, and the maximum control inclined depth has reached 680 m; this ore body is not seen on exploration Line 12. The No. 3 orebody is located in the middle and north of the mining area, with a total length of approximately 2800 m and a general thickness of 100 ~ 200 m; the deepest has reached 1600 m. The No. 4 orebody is distributed in the middle and north of the mining area, with a length of 1600 m.

**Figure 5:**
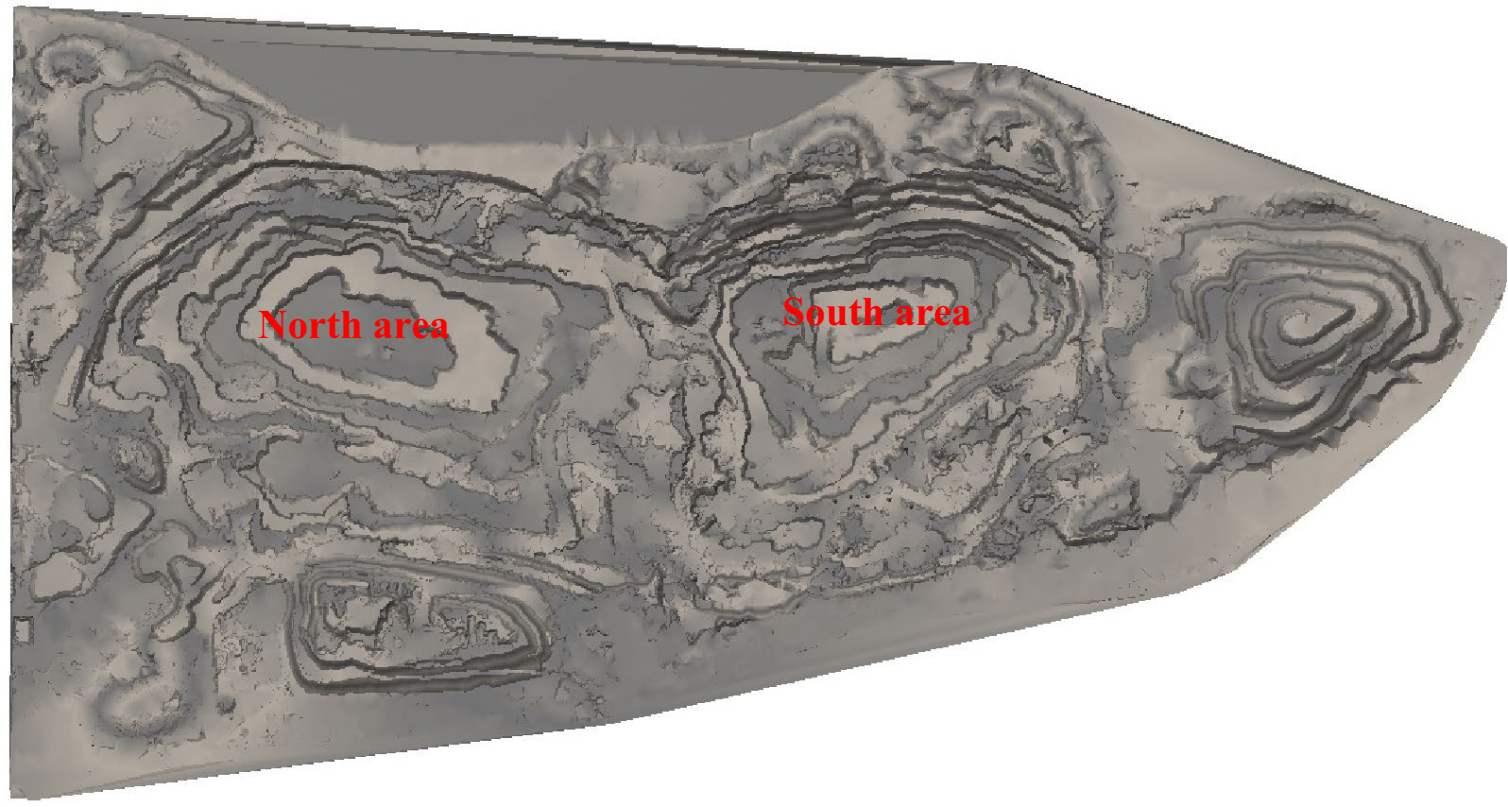
Mining status of pit.

The mining status of the pit is shown in Fig. 5. The northern area has been mined to  − 67 m, and the southern area has been mined to  − 112 m. The unit weight of ore in the pit is 3.28 t/m^3^, the unit weight of rock is 2.7 t/m^3^, and the unit weight of topsoil is 2.0 t/m^3^. The height of benches above the 67 m level is 12 m, and below is 15 m. The slope angle of the bench is 65°, the width of the safety platform is 8 m, and the width of the working platform is 45 m.

### Generation of discrete bodies

**Figure 6:**
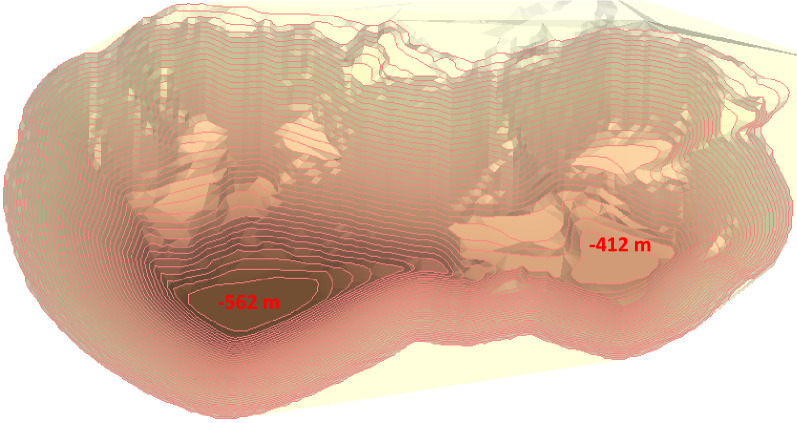
The ultimate pit of open pit mine.

Based on the ultimate pit optimization algorithm constructed by Xu et al.^109^, the ultimate pit obtained is shown in Fig. 6. The quantities of ore and rock in the pit are 584.617 million tons and 2183.859 million tons, respectively. The minimum mining depths in the north and south are − 562 m and − 412 m, respectively. The pit covers an area of 435.99 × 10^4^m^2^.

**Figure 7:**
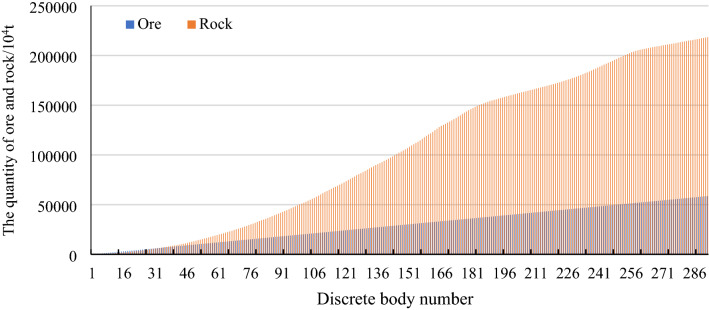
Discrete body sequence.

First, a series of wholly nested discrete bodies^1^ is generated according to a working slope angle of 24°, an ore increment of two million tons, and a cutoff grade of 25%, as shown in Fig. 7. A total of 292 discrete bodies are generated.

### Optimization parameter design

The production scheduling optimization of open pit mines needs to determine parameters including economic parameters (mining cost, processing cost, stripping cost, concentrate price, infrastructure investment base, infrastructure investment of each ton of ores, discount rate, and cost and price increase rate), technical parameters (mining recovery rate, processing recovery rate, waste rock mixing rate, and cutoff grade), geological parameters (mixed waste rock grade, average grade, and concentrate grade), constraint parameters (production capacity range, maximum quantity of annual mining and stripping, infrastructure period, and pit closure period). Table 1 lists the specific parameter values.

**Table 1:** The value of production scheduling optimization.

Mining cost (¥/t)	Stripping cost (¥/t)	Processing cost (¥/t)	Concentrate price (¥/t)	Lower limit of production capacity/10^4^t
72	9	105	900	800
Discount rate/%	Concentrate grade/%	Processing recovery rate/%	Pit closure period/a	Upper limit of production capacity/10^4^t
8	66	80	1	5000
Price increase rate/%	Cutoff grade/%	Mining recovery rate/%	Infrastructure period/a	Infrastructure investment base/10^4^ ¥
2.5	25	94	2	25,000
Cost increase rate/%	Waste rock grade/%	Waste rock mixing rate/%	infrastructure investment of each ton of ores( ¥/t)	
2	10	6	350	

### Analysis of production scheduling optimization results

**Figure 8:**
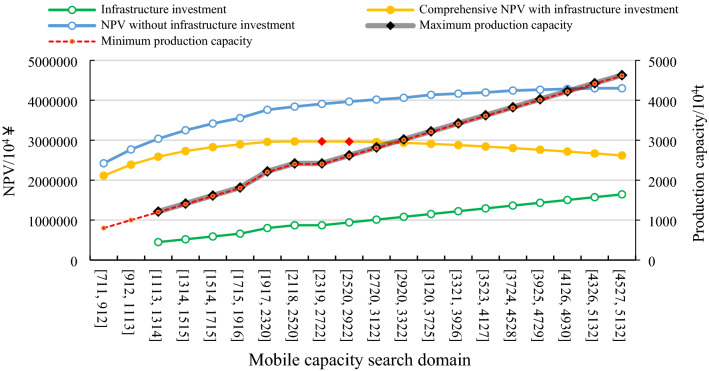
Results of optimal schemes in different capacity search domains.

Based on the discrete body sequence shown in Fig. 7, combined with the relevant parameter settings in Table 1, the optimization algorithm constructed above is used to optimize production scheduling. In the optimization process, it is considered that the infrastructure project is built according to the maximum production capacity of the mine. However, if the production capacity fluctuates significantly in the production process of the mine, the equipment will be idle. Therefore, considering that the actual annual ore production is less than 90% of maximum capacity, idle costs will be incurred; idle costs for one year are 7% of the investment. In Eq. (10), the search domain constraint (*x*_1_, *x*_2_, *x*_3_) is (33, 20, 16), and the obtained optimization results are shown in Fig. 8.

According to Fig. 8, the horizontal coordinate represents the range of the mobile capacity search domain, that is, the production capacity constraint range. For example, [711, 912] indicate that the production capacity is constrained between 7.11 million tons and 9.12 million tons. The enumeration method can be used within this production capacity range to determine all workable paths (meeting the production capacity constraint) and calculate NPV. Meanwhile, the maximum production capacity corresponding to each path can be found to calculate infrastructure investment. Finally, the path of maximum NPV within this constraint is obtained. At the same time, this is the best production scheduling, including mining sequence, production capacity and mining life. Since the lower limit of production capacity is 8 million tons (generally speaking, if the ore reserves in the ultimate pit exceed 500 million tons, the production capacity will not be less than 10 million tons) and the upper limit is 50 million tons, the mining life of large open pit mines will not be less than 10 years. Therefore, there are 20 mobile search domains ranging from 7.11 million tons to 51.32 million tons, with a minimum width of 2 million tons (a whole increment) and a maximum width of 8 million tons (four increments).

Through dynamic sorting and production scheduling optimization of 292 state variables (discrete bodies), 20 search domains and 3 optimization elements, a total of 76.4 billion paths were searched, which took 5 h and 20 min. According to the traditional search method, if the optimization is conducted directly in the production capacity [800, 5000], the optimal solution is the same, but a total of 2000 billion paths are searched, which takes a month.

**Figure 9:**
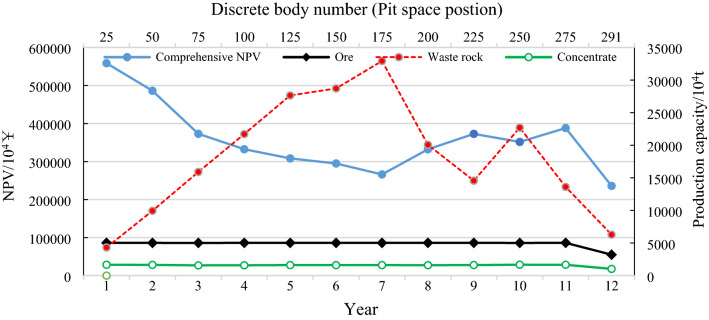
NPV maximum production scheduling without infrastructure investment.

According to the blue line in Fig. 8, when infrastructure investment is not considered, the greater the mine production capacity is, the greater NPV will be due to the reasons of scale efficiency and time value of capital. Therefore, in these 20 search domains, the maximum value of the total NPV of the mine is generated in the search domain [4527, 5132], which is 43.001 ¥ billion, and the production capacity of the best production scheduling (excluding production capacity during infrastructure and pit closure) is within this range. Figure 9 shows the production scheduling corresponding to the maximum NPV, including the annual NPV (principal Y-axis), ore quantity, waste rock quantity, concentrate quantity (secondary Y-axis), mining life (principal X-axis) and annual pit advancing position (discrete body number, secondary X-axis). Among them, the maximum production capacity is 50.31 million tons. The stripping peak occurred in the 5th to 7th years (276.409 million tons, 287.141 million tons and 32.9222 million tons, respectively), and the spatial position of the pit showed discrete body numbers 125, 150 and 175, respectively. The mining life is 12 years.

**Figure 10:**
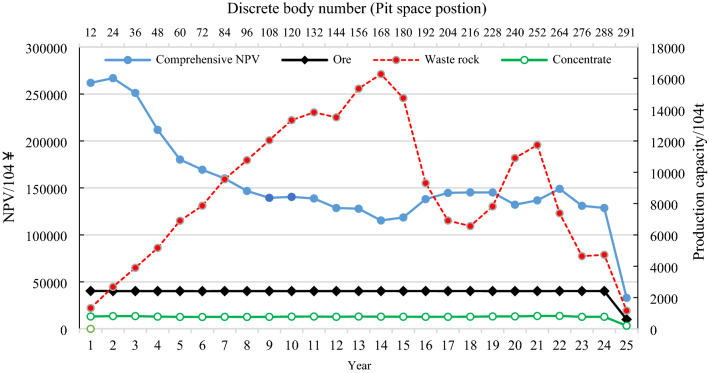
NPV maximum production scheduling with infrastructure investment.

When infrastructure investment is considered, since infrastructure investment positively correlates with production capacity, the greater the production capacity is, the greater the infrastructure investment. Therefore, with the increase in production capacity, the mine comprehensive NPV first increases and then decreases (as shown by the yellow line in Fig. 8). The maximum value of NPV occurs in the intersection area of search domains [2319, 2722] and [2520, 2922], which is 29.689 billion ¥. Figure 10 shows the production scheduling corresponding to this maximum NPV, including annual NPV (principal Y-axis), ore quantity, waste rock quantity, concentrate quantity (secondary Y-axis), mining life (principal X-axis), and annual pit advancing position (discrete body number, secondary X-axis). Furthermore, with a maximum production capacity of 24.2 million tons (infrastructure investment of 8.72 ¥ million) and a minimum production capacity of 24.04 million tons (excluding production capacity during infrastructure and pit closure), the peak of stripping occurred in the 9th to 15th years (120.447 million tons, 133.282 million tons, 138.258 million tons, 135.101 million tons, 153.425 million tons, 162.709 million tons and 147.378 million tons, respectively). The spatial position of the pit was shown as discrete body numbers 108, 120, 132, 144, 156, 168 and 180. The mining life is 25 years (Fig. 10).

**Figure 11:**
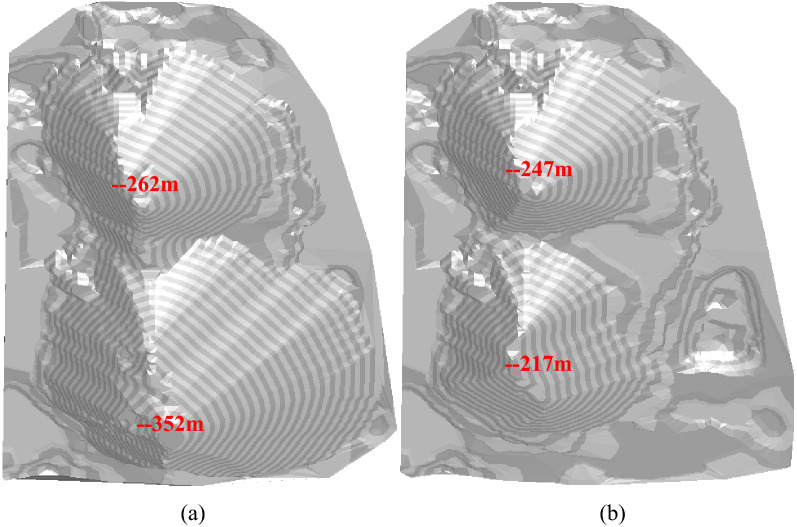
Pit status in the 5th year of the production scheduling with production capacity of 50.31 million tons (**a**) and 24.2 million tons (**b**) respectively.

Comparing Figs. 9 and 10, it can be seen that considering infrastructure investment in the optimization process of production scheduling has a great impact on mine production. The infinite expansion of production capacity is well constrained due to the consideration of infrastructure investment, and large quantities of rock stripping projects in the short term are balanced to avoid investment waste. The production capacity was reduced by 51.9%, the mining life was extended by 108.3%, and the mining sequence also changed greatly (for example, in the 5th year, the pit advancing position considering infrastructure investment is discrete body 60, while that not considering infrastructure investment is discrete body 125). The three-dimensional comparison of pit status in the 5th year is shown in Fig. 11.

As shown in Fig. 8, without infrastructure investment, the maximum production scheduling of NPV occurs in the intersection of search domains [4326, 5132] and [4527, 5132], which is 43.001 ¥ billion. The maximum production capacity of this scheduling in Fig. 9 is 50.31 million tons. According to Eq. (7) and parameter values in Table 1, it can be calculated that if the infrastructure is carried out following the scheduling, the infrastructure investment is 17.858 ¥ billion. Although infrastructure investment is not considered in the optimization process, infrastructure investment must be deducted according to the maximum production capacity of the optimal scheme. Therefore, the mine's final NPV is 25.142 ¥ billion. Under the condition of considering infrastructure investment, the maximum production scheduling of NPV (Fig. 10) occurs in the intersection area of search domains [2319, 2722] and [2520, 2922], which is 296.898 ¥ billion (Fig. 8). It can be seen that the overall economic benefit of the mine can be significantly increased by 18% when infrastructure investment is considered in the process of production scheduling optimization.

On one hand, if the production capacity of a mine fluctuates too much, it will increase the difficulty of management; on the other hand, it will also increase the cost investment. According to Fig. 8, the solid gray line and dotted red line corresponding to the subcoordinates represent the optimal production scheduling's maximum and minimum production capacity, respectively, in the corresponding search domain, regardless of the production capacity of infrastructure and pit closure. Moreover, it can be seen that the two lines almost overlap, and the fluctuation of production capacity of all optimal production scheduling in all search domains does not exceed 1.4%, which indicates that the "mobile capacity search domain" can not only ensure that the optimal production capacity is not omitted (the intersection part of two adjacent search domains) but can also effectively control the fluctuation of mine production capacity. Simultaneously, in the optimization process, the idle cost will be generated if the production capacity fluctuates greatly; therefore the disorderly expansion of production capacity will also play a constraint role in the process of economic optimization.

## Conclusion

In view of the problems in the process of production scheduling optimization of open pit mines, such as low operation efficiency caused by a large amount of calculation, disorderly expansion of production capacity caused by the scale effect, and difficulties in optimizing the three elements at the same time due to the interaction relationship, taking the maximum comprehensive NPV as the objective function, the mobile capacity search domain method is proposed to improve the operation efficiency. Furthermore, the infrastructure investment function based on the maximum production capacity is constructed to restrict the production capacity, the facility idle threshold is designed to reduce the fluctuation range of production capacity, and the enumeration method is used to evaluate all workable paths to realize the simultaneous optimization of the production capacity, mining sequence and mining life of open pit mines. The results show the following:

1. The mobile capacity search domain method can intelligently calculate the optimization space according to the reserve scale and the lower limit of productivity and automatically design the capacity domain range, thus effectively reducing the number of search paths and improving the operation efficiency by 200 times. This method can significantly reduce the operation scale for open pit mines with a large number of discrete bodies, especially for the high-precision production scheduling design of large-scale open pit mines (the smaller the increment between discrete bodies is, the higher the precision), and has significant practical value.
2. In the process of production scheduling optimization, the consideration of infrastructure investment significantly influences production capacity, mining sequence and mining life. Infrastructure investment has effectively restrained the phenomenon of disorderly expansion of production capacity. Due to the scale effect, the life of mines has been prolonged by 1.1 times, which is more in line with mine production design specifications. In addition, considering the idle cost of infrastructure investment, the fluctuation range of production capacity is less than 1.4%. The peak of waste rock stripping is delayed for 4 years, and there is a relatively stable peak of waste rock stripping for 7 years, which balances large quantities of waste rock stripping projects in the short term and is convenient for mine management and cost savings.
3. When the infrastructure investment, which is set as a function of the maximum production capacity, is incorporated into the production scheduling optimization, the comprehensive NPV obtained is higher than that considered in the production scheduling optimization and infrastructure investment accounting. The scale effect of mines is only suitable for a certain range of production capacity. When the production capacity is small, with the increase in production capacity, the scale effect is reflected in reducing the unit production cost of the mine. When the production capacity reaches a certain level, due to the high infrastructure construction investment, the increase in unit cost is higher than the impact of the scale effect, which makes the overall economic benefits of the mine decline. The study shows that considering infrastructure investment in the process of production scheduling optimization can increase the overall economic benefit of the mine by 18%, which is very significant.

This study will continue to explore the functional relationship between mine production capacity and infrastructure investment. This paper is based on the data of multiple mines to synthesize a linear relationship, but the statistical samples are limited. From the perspective of economy and efficiency, infrastructure investment and production capacity are also contents that need to be optimized. At the same time, what needs to be further studied is the impact of different reserve sizes (the size of ultimate pit) on the overall economic benefits of the mine. This paper optimizes the production scheduling based on the given ultimate pit. For various ultimate pits, due to different ore and rock quantities, and spatial forms, the production scheduling will also be different, resulting in different economic benefits. What kind of reserve sizes and corresponding production scheduling are the best needs further scientific exploration.

## Supplementary Information

Supplementary Information.

## Data Availability

All data generated or analysed during this study are included in this published article and supplementary file.
